# High Fat Diet Exposure during Fetal Life Enhances Plasma and Hepatic Omega-6 Fatty Acid Profiles in Fetal Wistar Rats

**DOI:** 10.3390/nu7095337

**Published:** 2015-08-28

**Authors:** Marlon E. Cerf, Johan Louw, Emilio Herrera

**Affiliations:** 1 Diabetes Discovery Platform, South African Medical Research Council, P.O. Box 19070, Tygerberg, Cape Town 7505, South Africa; E-Mail: johan.louw@mrc.ac.za; 2 Department of Chemistry and Biochemistry, University of San Pablo-CEU, Ctra. Boadilla del Monte km 5.3, Madrid 28668, Spain; E-Mail: eherrera@ceu.es

**Keywords:** fatty acid analysis, fetal programming, metabolic disease

## Abstract

Pregnant rats were fed a high fat diet (HFD) for the first (HF1), second (HF2), third (HF3) or all three weeks (HFG) of gestation. Maintenance on a HFD during specific periods of gestation was hypothesized to alter fetal glycemia, insulinemia, induce insulin resistance; and alter fetal plasma and hepatic fatty acid (FA) profiles. At day 20 of gestation, fetal plasma and hepatic FA profiles were determined by gas chromatography; body weight, fasting glycemia, insulinemia and the Homeostasis Model Assessment (HOMA-insulin resistance) were also determined. HF3 fetuses were heaviest concomitant with elevated glycemia and insulin resistance (*p* < 0.05). HFG fetuses had elevated plasma linoleic (18:2 *n*-6) and arachidonic (20:4 *n*-6) acid proportions (*p* < 0.05). In the liver, HF3 fetuses displayed elevated linoleic, eicosatrienoic (20:3 *n*-6) and arachidonic acid proportions (*p* < 0.05). HFG fetuses had reduced hepatic docosatrienoic acid (22:5 *n*-3) proportions (*p* < 0.05). High fat maintenance during the final week of fetal life enhances hepatic omega-6 FA profiles in fetuses concomitant with hyperglycemia and insulin resistance thereby presenting a metabolically compromised phenotype.

## 1. Introduction

Fetal metabolism, and consequently fetal growth, directly depends on the nutrients crossing the placenta, and therefore the mother adapts her metabolism to support this continuous draining of substrates [1].

Fatty acids (FAs) are important for fetal growth and development. Specifically, linoleic acid (18:2 *n*-6) and α-linolenic acid (ALA, 18:3 *n*-3) are the essential fatty acids (EFAs), which together with their long-chain polyunsaturated FA (LCPUFA) derivatives are essential for fetal and postnatal development. The fetus obtains both EFAs and LCPUFAs from maternal circulation by transfer across the placenta [2,3].

High intakes of *n*-3 FAs solely during early pregnancy in rats have beneficial long-term consequences in their offspring, reducing the age-related decline in insulin sensitivity in male offspring [4].

A high fat diet (HFD) was shown to contribute to the development of obesity, insulin resistance, type 2 diabetes and the impairment of the glucose signaling system in the beta cells [5,6,7,8]; in neonates, these effects were dependent on the period of fetal life when the HFD was administered [5]. In the present study, we investigated, in fetal rats, how exposure to a HFD during specific weeks of fetal life influences plasma and hepatic FA profiles, glycemia, insulinemia and insulin resistance.

## 2. Experimental Section

**Figure 1:**
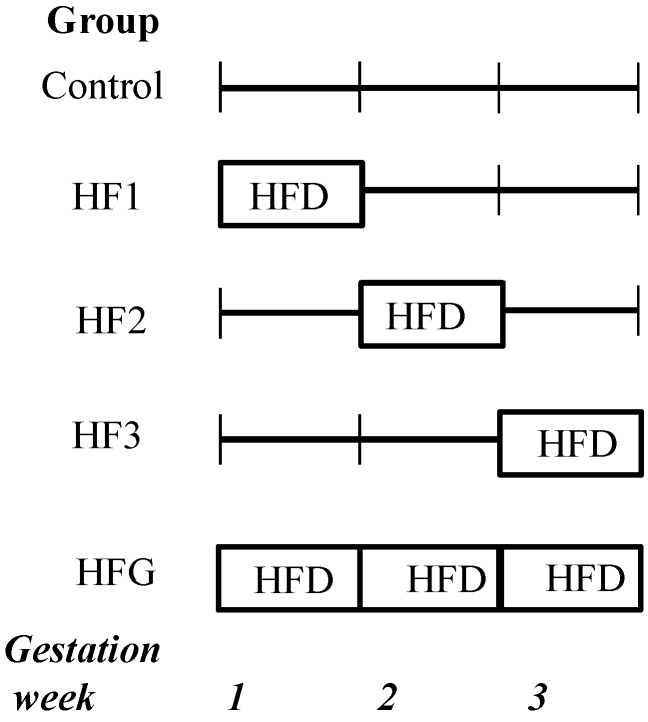
Experimental groups. The fetuses were maintained (through their mothers’ nutrition) on a high fat diet (HFD) for either the first week (HF1), second week (HF2), third week (HF3) or all 3 weeks (HFG) of fetal life.

Institutional ethical approval was obtained before the experiments commenced. Wistar rats were maintained as previously described [5]. Briefly, pregnant mothers were fed a HFD during specific periods of gestation. The experimental groups are displayed in Figure 1. Although the HFD was administered to the mothers, their fetal offspring were subsequently maintained on the respective gestational diets. In the present study, 20-day-old fetuses maintained on a HFD for the first (HF1), second (HF2), third (HF3) or all three (HFG) weeks of fetal life were studied. Specifically, HF1 fetuses were maintained on a HFD from embryonic day (e) 0–7, HF2 fetuses from e8–14, HF3 fetuses from e15–20 and HFG fetuses from e0–20. When HF1, HF2 and HF3 fetuses were not maintained on a HFD, they were instead maintained on a standard laboratory diet for the remainder of fetal life, e.g., HF1 fetuses were maintained on a HFD for the first week of fetal life and a control diet for weeks 2 and 3 of fetal life (Figure 1). Fetuses maintained on a standard laboratory diet throughout represented the control group. The standard laboratory diet (control) (Epol, South Africa) comprised 10% fat, 15% protein and 75% carbohydrate (2.6 kcal/g). The HFD contained 40% fat, 14% protein and 46% carbohydrate (2.06 kcal/g). The HFD predominantly comprised saturated FAs (myristic, palmitic and stearic acid) and the mono-unsaturated FA, oleic acid, derived from animal fat with carbohydrates mainly derived from starch to mimic a westernized diet. The control diet was a standard commercial rodent laboratory diet.

Mothers were maintained on a HFD for specific periods of gestation and their 20-day-old fetal offspring were studied. Therefore fetuses were maintained (through their mothers’ nutrition) on HFD for either the first (HF1), second (HF2), third (HF3) or all 3 weeks (HFG) of fetal life. Control fetuses were maintained on a standard commercial rodent laboratory diet.

At day 20 of pregnancy, mothers were euthanized after a 3 h fast. After removal from the uterus, fetuses were weighed and decapitated and the trunk blood was used for FA analysis. Whole liver was collected, weighed and stored at −80 °C until analysis. Blood was collected on ice-chilled tubes containing 1 g/L of Na_2_-EDTA and pooled per litter from each mother. The pooled fetal blood was centrifuged and plasma aliquots were stored at −20 °C until analysis.

Blood glucose (glucometer, Precision QID, MediSense, Oxfordshire, UK) and serum insulin (rat insulin radio-immunoassay (RIA) kit, Linco Research, St. Charles, MO, USA) concentrations were measured. The Homeostasis Model Assessment (HOMA-insulin resistance) was calculated (fasting plasma glucose (mmol/L) × fasting serum insulin (mU/L)/22.5)).

For FA analysis, nonadecenoic acid (19:1; Sigma-Aldrich, Madrid, Spain) was added as the internal standard to fresh aliquots of frozen plasma and liver which were used for lipid extraction and purification [9]. The final lipid extract was evaporated under vacuum and the residue suspended in methanol/toluene followed by methanolysis in the presence of acetyl chloride at −80°C for 2.5 h as previously described [10]. FA methyl esters were separated and quantified on a gas chromatograph (Autosystem, Perkin-Elmer, Madrid, Spain) with a flame ionization detector and a 20 m Omegawax capillary column (internal diameter 0.25 mm). Nitrogen was used as carrier gas and the FA methyl esters were compared to purified standards (Sigma-Aldrich). Quantification of the FAs in the samples was performed as a function of the corresponding peak areas and compared to the internal standard.

One-way analysis of variance (ANOVA) and Bonferroni’s post-test were applied with data reported as means ± standard error of the means (SEM) and significance established at *p* < 0.05.

## 3. Results

### 3.1. Anthropometry

As shown in Table 1, the HF3 fetuses had higher body weights relative to the other fetuses. HF2 fetuses had lower liver weights compared to the control, HF3 and HFG fetuses; after adjustment for body weight, lower HF2 fetal liver weights persisted relative to all the groups (Table 1).

**Table 1:** Fetal anthropometry and metabolic parameters of 20-day-old rat fetuses.

	Control	HF1	HF2	HF3	HFG
Weight (g)	3.19 ± 0.06	3.23 ± 0.07	3.22 ± 0.05	3.79 ± 0.11 *^,†,‡,ᴧ^	3.20 ± 0.07
Liver weight (mg)	246.7 ± 12.69	196.0 ± 11.47	144.0 ± 16.85 *^,§,ᴧ^	253.7 ± 20.03	218.7 ± 10.81
Adjusted liver weight (mg/100 g)	7.26 ± 0.24	6.23 ± 0.38	4.31 ± 0.50 *^,†,§,ᴧ^	6.67 ± 0.43	6.95 ± 0.25
Blood glucose (mmol/L)	2.54 ± 0.12	3.16 ± 0.16	2.97 ± 0.23	4.99 ± 0.56 *^,†,‡,ᴧ^	3.06 ± 0.20
Serum insulin (pM)	48.24 ± 13.57	66.59 ± 11.50	62.68 ± 13.33	86.10 ± 22.96	75.72 ± 16.54
HOMA-insulin resistance	1.09 ± 0.26	1.84 ± 0.30	1.68 ± 0.42	3.59 ± 0.13*	2.42 ± 0.92

The fetuses were maintained (through their mothers’ nutrition) on a high fat diet (HFD) for either the first week (HF1), second week (HF2), third week (HF3) or all 3 weeks (HFG) of fetal life. Values are means ± standard error of the means (SEM). *n* = 31–59 per group, but *n* = 3–6 for serum insulin and Homeostasis Model Assessment (HOMA)-insulin resistance (due to pooling). Bonferroni’s test was used to determine differences between groups after ANOVA. HF = high fat. Numerals refer to the week of maintenance on a high fat diet. * *p* < 0.05 *vs.* control, ^†^ *p* < 0.05 *vs.* HF1, ^‡^ *p* < 0.05 *vs.* HF2, ^§^ *p* < 0.05 *vs.* HF3, ^ᴧ^ *p* < 0.05 *vs.* HFG.

### 3.2. Metabolic Parameters

The HF3 fetuses had higher glucose concentrations relative to the other fetuses (Table 1). However, insulin concentrations did not differ amongst the groups (Table 1). Further, the HF3 fetuses were also insulin resistant evident by their higher HOMA-insulin resistance values compared to the control fetuses (Table 1).

### 3.3. Plasma and Hepatic FA Profiles

Changes in the FA profile in the maternal diet may affect the availability of certain FAs in fetal plasma. HFG fetuses had enhanced plasma omega-6 FA profiles. Specifically, plasma linoleic acid was higher in HFG fetuses relative to control, HF1 and HF2 fetuses (Table 2). Further, plasma dihomo-gamma-linolenic acid (DGLA; 20:3 *n*-6) was higher in HFG fetuses compared to HF1 fetuses. In addition, plasma arachidonic acid (20:4 *n*-6) was higher in HFG fetuses compared to control and HF1 fetuses (Table 2).

**Table 2:** Plasma fatty acid profiles (%) of 20-day-old rat fetuses maintained on a high fat diet.

Fatty Acid	Control	HF1	HF2	HFG
Saturated				
14:0	1.59 ± 0.17	2.47 ± 0.40	1.86 ± 0.17	2.20 ± 0.17
16:0	27.47 ± 0.57	32.34 ± 3.59	31.77 ± 0.99	25.90 ± 0.54
18:0	11.43 ± 0.60	12.49 ± 0.58	11.83 ± 0.49	11.20 ± 0.60
22:0	0.84 ± 0.09	0.81 ± 0.19	0.82 ± 0.05	0.75 ± 0.11
24:0	0.84 ± 0.14	0.72 ± 0.01	0.65 ± 0.09	0.71 ± 0.18
Monounsaturated				
14:1	0.62 ± 0.28	0.71 ± 0.08	1.29 ± 0.30	0.96 ± 0.22
16:1 (*n*-7)	5.42 ± 0.52	5.00 ± 0.52	5.61 ± 0.41	5.27 ± 0.42
18:1 (*n*-9)	25.55 ± 1.23	25.23 ± 0.39	23.65 ± 0.49	26.15 ± 0.90
Omega-3				
20:5 (*n*-3)	1.34 ± 0.45	2.03 ± 1.39	1.08 ± 0.37	1.05 ± 0.63
22:5 (*n*-3)	0.58 ± 0.27	-	0.13 ± 0.05	0.69 ± 0.35
22:6 (*n*-3)	8.99 ± 0.68	5.44 ± 1.15	7.19 ± 1.10	5.57 ± 0.98
Omega-6				
18:2 (*n*-6)	8.10 ± 0.35	6.54 ± 0.47	7.23 ± 0.42	9.87 ± 0.29 *^,†,‡^
18:3 (*n*-6)	0.38 ± 0.03	0.32 ± 0.04	0.41 ± 0.03	0.48 ± 0.04
20:3 (*n*-6)	0.45 ± 0.10	0.38 ± 0.12	0.50 ± 0.04	0.76 ± 0.03 ^†^
20:4 (*n*-6)	5.84 ± 0.39	5.05 ± 0.94	5.97 ± 0.62	8.41 ± 0.32 *^,†^

The fetuses were maintained (through their mothers’ nutrition) on a high fat diet (HFD) for either the first (HF1), second (HF2), third (HF3) or all 3 weeks (HFG) of fetal life. Values are means ± standard error of the means (SEM). *n* = 3–4 per group. *n* = 2 for HF3 which was excluded from analyses. Bonferroni’s test was used to determine differences between groups after one-way analysis of variance (ANOVA). HF = high fat; Numerals refer to the week of maintenance on a high fat diet. * *p* < 0.05 *vs.* control, ^†^ *p* < 0.05 *vs.* HF1, ^‡^ *p* < 0.05 *vs.* HF2 − *n* = 2 for HF1.

As shown in Table 3, the proportion of different FAs in fetal liver remained relatively constant. However, specific omega-6 FAs were elevated in HF3 fetuses: linoleic (compared to control and HF2 fetuses), eicosatrienoic (compared to HF1 fetuses) and arachidonic acid (compared to control fetuses; Table 3). Further, hepatic docosapentaenoic acid (DPA; 22:5 *n*-3) was lower in HFG fetuses compared to control and HF3 fetuses (Table 3).

**Table 3:** Hepatic fatty acid profiles (%) of 20-day-old rat fetuses exposed to a maternal high fat diet.

Fatty Acid	Control	HF1	HF2	HF3	HFG
Saturated					
14:0	1.03 ± 0.20	0.93 ± 0.09	1.00 ± 0.07	1.39 ± 0.16	1.22 ± 0.15
16:0	24.46 ± 0.92	23.15 ± 0.43	23.62 ± 0.59	24.20 ± 0.90	23.75 ± 0.50
18:0	15.40 ± 0.40	15.57 ± 0.37	14.85 ± 0.17	15.20 ± 0.43	14.55 ± 0.21
20:0	0.31 ± 0.05	0.30 ± 0.04	0.26 ± 0.01	0.28 ± 0.02	0.32 ± 0.04
Monounsaturated					
15:1	0.49 ± 0.05	0.33 ± 0.09	0.34 ± 0.07	0.36 ± 0.06	0.36 ± 0.06
16:1 (*n*-7)	4.20 ± 0.37	3.82 ± 0.31	3.93 ± 0.13	3.43 ± 0.23	4.03 ± 0.11
18:1 (*n*-9)	21.01 ± 0.70	20.09 ± 0.77	21.35 ± 0.79	19.03 ± 1.31	22.44 ± 0.67
20:1 (*n*-9)	0.21 ± 0.07	0.24 ± 0.03	0.24 ± 0.01	0.23 ± 0.03	0.23 ± 0.03
Omega-3					
20:5 (*n*-3)	0.83 ± 0.10	0.70 ± 0.08	0.57 ± 0.03	0.65 ± 0.09	0.69 ± 0.10
22:5 (*n*-3)	0.40 ± 0.03	0.37 ± 0.06	0.34 ± 0.03	0.41 ± 0.04	0.24 ± 0.01 *^,§^
22:6 (*n*-3)	12.64 ± 0.91	13.00 ± 0.96	12.01 ± 0.56	9.99 ± 1.21	8.81 ± 0.51
Omega-6					
18:2 (*n*-6)	7.24 ± 0.30	7.86 ± 0.53	8.06 ± 0.25	10.16 ± 0.79 *^,‡^	8.79 ± 0.30
20:2 (*n*-6)	0.22 ± 0.03	0.25 ± 0.05	0.24 ± 0.02	0.25 ± 0.01	0.23 ± 0.02
20:3 (*n*-6)	0.83 ± 0.03	0.79 ± 0.03	0.87 ± 0.06	1.03 ± 0.03 ^†^	0.99 ± 0.05
20:4 (*n*-6)	8.68 ± 0.33	10.53 ± 0.46	10.21 ± 0.26	10.95 ± 0.52*	10.65 ± 0.72
22:4 (*n*-6)	0.40 ± 0.01	0.43 ± 0.01	0.47 ± 0.02	0.50 ± 0.05	0.48 ± 0.04

The fetuses were maintained (through their mothers’ nutrition) on a high fat diet (HFD) for either the first week (HF1), second week (HF2), third week (HF3) or all 3 weeks (HFG) of fetal life. Values are means ± standard error of the means (SEM). *n* = 3–5 per group. Bonferroni’s test was used to determine differences between groups after one-way analysis of variance (ANOVA). HF = high fat. Numerals refer to the week of maintenance on a high fat diet. * *p* < 0.05 *vs.* control, ^†^ *p* < 0.05 *vs.* HF1, ^‡^ *p* < 0.05 *vs.* HF2, ^§^ *p* < 0.05 *vs.* HF3.

## 4. Discussion

The hyperglycemia and insulin resistance in fetuses maintained on a HFD for the final week of fetal life concomitant with increased hepatic omega-6 FA proportions were the main findings. During the final week of fetal life the endocrine pancreas differentiates into functional islet cells specialized for the maintenance of glucose homeostasis. Fetal programming refers to intrauterine stimuli or insults (such as the insult of maintenance on a HFD) that have immediate, transient or durable effects. With the fetal high fat programming insult during this critical developmental window, viz., late fetal life, islet cell, and specifically beta cell, development coincides with the HFD insult. We have previously shown that neonates maintained on a HFD throughout fetal life had compromised beta cell development and function [11,5]. Fetal high fat programming may therefore contribute to the hyperglycemia and insulin resistance in fetuses maintained on a HFD for the final week of fetal life.

In Westernized society, diets contain omega-6 to omega-3 ratios that far exceed the recommended ratio of 1:1; this increased ratio is believed to contribute to the global increase in metabolic disease.

In rodents, from e15 up to birth and into postnatal life, the maturation and growth of the liver occurs [12,13] with liver expansion of 84-fold, from e13.5–20.5, evident by the hepatoblasts undergoing 8-doublings [14,15,16]. Hence this critical hepatic developmental period of rapid and significant expansion overlaps with the administration of the HFD to HF3 neonates (exposed to a HFD from e15–20). With an increase of the precursor, linoleic acid, in the HF3 fetal livers, more substrate was available for conversion into arachidonic acid to help meet the fetal growth demands. FA elongation and desaturation are two key metabolic routes for the synthesis of saturated, monounsaturated and polyunsaturated FAs [17]. The elevated hepatic eicosatrienoic acid provided further evidence of lipogenesis along the omega-6 pathway. This also suggested activation of delta-6 desaturase, elongase and delta-5 desaturase to facilitate fetal hepatic lipogenesis. However, this requires the application of assays to assess enzymatic activities followed by Western blot analyses. However, enzyme activity and expression should be studied in older progeny due to low elongase and desaturase expression in the fetal liver [18]. The increase in hepatic omega-6 FA in fetuses maintained on a HFD for the final week of fetal life appears to contribute to their compromised metabolic phenotype. The exact role of the elevated hepatic omega-6 FAs in inducing these metabolically compromised fetuses remains to be elucidated.

DPA (22:5 *n*-3) is an elongation metabolite of eicosapentaenoic acid (EPA; 20:5 *n*-3) and an underexplored omega-3 FA. Supplementation of liver cells with DPA down-regulated the expression levels of key genes and proteins involved in FA synthesis [19]. The reduced DPA *n*-3 proportions in the livers of fetuses maintained on a HFD throughout fetal life may therefore result in higher expression profiles of FA synthesis factors.

The elevated omega-6 plasma FAs in HFG fetuses to some extent mimicked the hepatic FA profiles of HF3 fetuses. The EFAs, linoleic and alpha-linoleic acid are supplied by the diet, and their LCPUFA derivatives, play a critical role in fetal development [1]. Linoleic acid’s metabolite, arachidonic acid, is essential for neonatal growth and development [20] which suggests that postnatal growth in these HF3 fetuses may be accelerated. Both fetuses and neonates are dependent upon the supply of preformed LCPUFAs from their mothers, which is obtained from maternal circulation [21]. An enhancement in arachidonic acid and docosahexaenoic acid (DHA; 22:6 *n*-3) in fetal circulation is called magnification and infers their effective transfer throughout the placenta [22]. The ratio of the proportions of arachidonic acid to linoleic acid is higher in fetal serum than in maternal serum [23], which suggests the preferential transfer of arachidonic acid through the placenta [24]. In all of the groups, the ratios of arachidonic acid to linoleic acid were elevated in the fetal plasma relative to the maternal plasma (data not shown) reflecting the preferential transfer of arachidonic acid through the placenta [24]. Despite the pooling of fetal litter plasma samples to yield sufficient volumes for analyses, the plasma sample number was too low for HF3 fetuses. Hence no HF3 fetal plasma FA profiles were determined which was a constraint. In summary, fetuses maintained on a HFD throughout fetal life had elevated plasma linoleic and arachidonic acid proportions concomitant with elevated plasma arachidonic acid to linoleic acid ratios in fetuses relative to mothers which suggested preferential placental transfer of arachidonic acid to sustain fetal and postnatal growth.

DGLA (20:3 *n*-6) is metabolized to the anti-inflammatory eicosanoid, prostaglandin (PG) E1, via the cyclooxygenase (COX) pathway [25] and was recently reported to be positively correlated to increased type 2 diabetes risk [26]. Although plasma DGLA proportions were increased in HFG fetuses relative to HF1 fetuses, the increase in this rare FA may not have any biological relevance [26].

Liver mass and its later function are essentially set during fetal development which is regulated by the intrauterine environment [27]. Disease risk is amplified by a greater mismatch between the prenatally predicted and actual adult environments [28]. Epidemiological data imply that hepatic organogenesis is susceptible to nutritional reprogramming and that impaired liver development in utero can result in durable functional consequences on disease risk later in life [27]. From e8–14 hepatic fate is specified, followed by gut tube formation, the liver domain relocating to the mid-gut followed by the liver diverticulum expanding into a liver bud [12,13]. These major liver development processes coincide with the HF2 neonates who had low liver weights. Hence the HFD administration during mid fetal life, i.e., e8–14, appeared to stunt liver development. The reduced liver weights in fetuses maintained on a HFD for the second week of fetal life may render them susceptible to metabolic disease.

Although we found altered fetal plasma and hepatic FA profiles in some offspring, our study has several limitations. Varying FA abundance can affect processes such as inflammation, angiogenesis and insulin sensitivity [29]. Early life hepatic fat accumulation is an early manifestation of non-alcoholic fatty liver disease (NAFLD) and an independent pathophysiological event that potentiates postnatal metabolic liver disease [30]. Therefore hepatic inflammation, steatosis and disrupted insulin signaling potentially contribute to the metabolically compromised phenotype that presented in the HF3 fetuses. Unfortunately FA analyses were conducted on all the frozen fetal liver samples, with no additional samples available for further investigation to reinforce our findings. Moreover, stratifying the study according to gender would likely have revealed gender-specific differences. The role of the elevated plasma and hepatic omega-6 FAs also remains to be fully elucidated.

## 5. Conclusions

Fetuses maintained on a HFD solely for the final week of fetal life were hyperglycemic and insulin resistant concomitant with enhanced hepatic omega-6 FA proportions, viz., linoleic, eicosatrienoic and arachidonic acid. These events may reflect enhanced omega-6 lipogenesis in response to the compromised metabolic phenotype.
